# Argentine ant chemical profiles vary by location on the Stanford University campus

**DOI:** 10.17912/micropub.biology.001475

**Published:** 2025-02-21

**Authors:** Aayesha Nangia, Mabel Gonzalez, Lauren A. O'Connell, Katherine Fiocca

**Affiliations:** 1 Dolores Huerta Middle School, San Jose, California, United States; 2 BioRETS INSPIRE Program, Stanford University, Stanford, California, United States; 3 Department of Biology, Stanford University, Stanford, California, United States

## Abstract

The Argentine ant ( *Linepithema humile* ) is an invasive ant species found across California. Many invasive ant species, including the Argentine ant, can use chemical defense compounds to ward off predators or compete with native ant species, which aids in invasive spread. Previously, Stanford undergraduate researchers found variation in the potency of Argentine ant chemical profiles (collected in varying locations on campus) in repelling *Caenorhabditis elegans* during chemotaxis assays. Here, we asked if variation in Argentine ant chemical extracts was related to collection location on the Stanford campus. We collected Argentine ants from five different locations and analyzed differences in their chemical profiles. Using gas chromatography coupled to mass spectrometry, we found variation in ant chemical profiles based on location collected, where many metabolites contributed to these differences. Five of these compounds were successfully annotated, including pyrazines that are known to have a repulsive function in insects. This work highlights the importance of sampling location impacting naturally-derived ant compounds and the importance of this variable in future chemotaxis assays in the classroom.

**Figure 1. Chemical profiles vary by Argentine ant collection location on the Stanford University campus:**
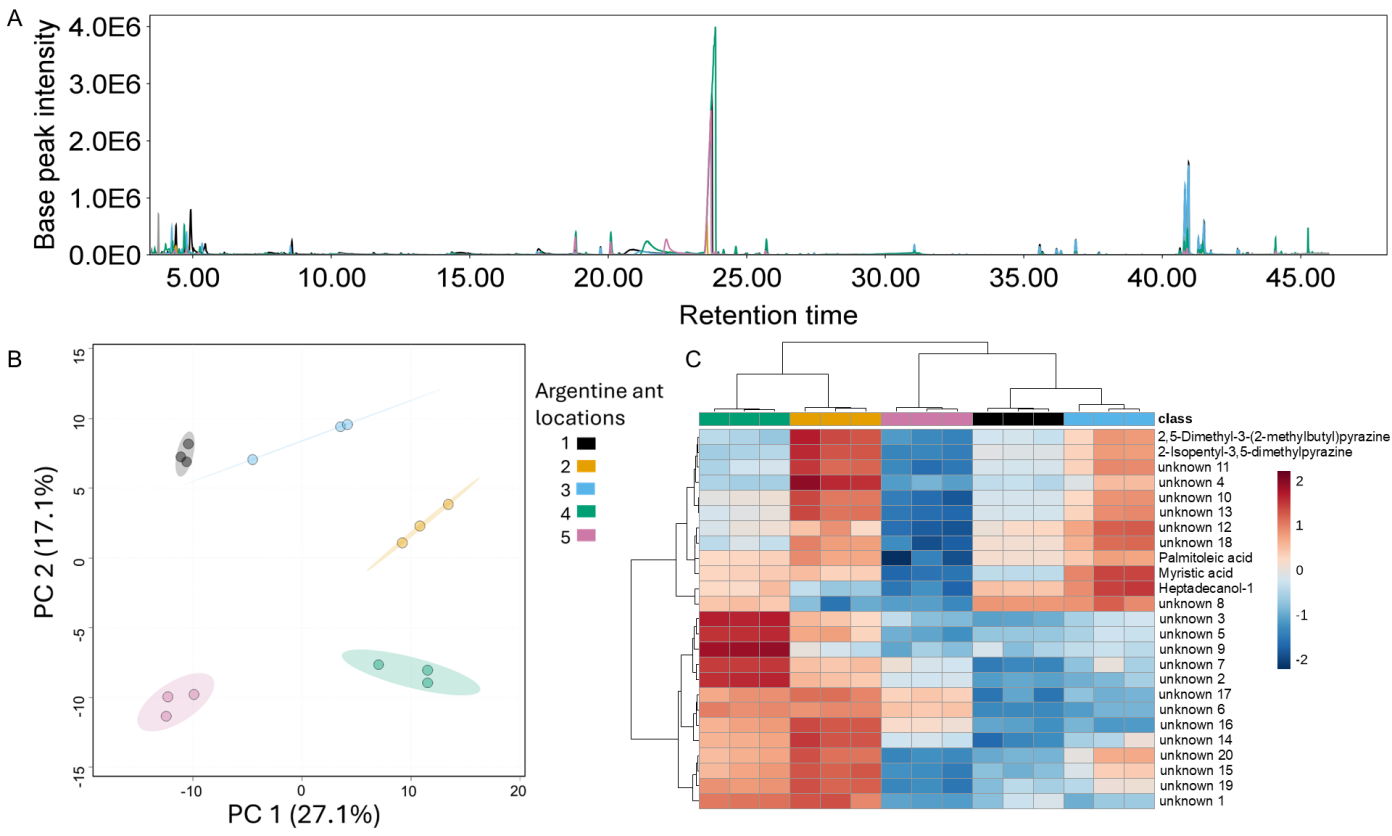
**(A)** Representative total ion chromatogram (TIC) of each collection location overlaid, with retention time in minutes and intensity of each feature after gas chromatography / mass spectrometry analysis. **(B)** Principal component analysis of Argentine ant chemical profiles collected in five different locations on campus. Each color corresponds to a collection location. **(C)** Heat map of top 25 metabolites contributing to the chemical differentiation of Argentine ants collected in five locations.

## Description

As one of the most invasive species across the globe, the Argentine ant ( *Linepithema humile* ) has found success in displacing native ants in Mediterranean climates. They outcompete native ants for resources and spread over large distances especially near human disturbance (Gordon and Heller, 2014; Helanterä, 2022; Salado et al., 2023) . Their environmental impact is well documented, including their economic significance (Angulo et al., 2022) , and disruption of native flora and fauna (Blancafort and Gómez, 2005; Bolger et al., 2000). Additionally, Argentine ants produce a defense compound (containing iridomyrmecin and dolichodial) that can irritate and deter competing ants, as well as some vertebrates like juvenile amphibians (Alvarez-Blanco et al., 2021; Welzel et al., 2018). It is unknown, however, what factors may impact variability of defensive compounds in Argentine ants, but colony location, among other factors, has been hypothesized to play an important role (Salado et al., 2023) .

Using chemotaxis assays with the nematode *Caenorhabditis elegans* provides a simple classroom model to explore chemosensation and its neural underpinnings, especially related to natural products and their behavioral impact on other taxa (Bargmann, 2006; Corsi et al., 2015) . Students in Stanford University’s BIO161 Organismal Biology Laboratory course previously asked whether chemical extracts from Argentine ants could invoke a chemotaxis response in *C. elegans* . Some experiments indicated Argentine ant extract can repel *C. elegans* (Alfonso et al., 2023) , while others suggested *C. elegans* does not respond (Lopez et al., 2024) . Given the variability in experimental outcomes, we hypothesized that chemical profiles of Argentine ants vary by sampling location on campus, providing a possible explanation for variability in *C. elegans* chemotaxis response for future undergraduate student experiments.

We collected workers from five locations of Argentine ants on the Stanford University campus and analyzed their chemical profiles using gas chromatography coupled to mass spectrometry. A representative total ion chromatogram plotted in Figure 1A shows that compounds extracted included highly volatile and semivolatile compounds. We tested whether Argentine ants differed in chemical profiles and found significant differences based on sampling location (PERMANOVA: F= 69.412, R ^2^ = 0.96524, p-value = 0.001; Figure 1B ). We then compared the normalized relative abundance of the top 25 metabolites contributing to this differentiation in the dataset and found that similarities between ant samples clustered by location ( Figure 1C ; Table 1), supporting the idea that variation in chemical profiles of Argentine ants are determined by geographic location. Previous sampling of a protected area nearby suggested that dispersal of Argentine ants is limited, where workers may mix within colonies of 100 meters or less, but otherwise colonies are genetically distinct from one another (Gordon and Heller, 2014; Ingram and Gordon, 2003) . Although not measured in this study, the impact of colony identity and relatedness on workers collected in geographically close (<100 m) sampling locations may be important to consider.

From the top 25 metabolites, five could be annotated, including two pyrazines (Table 1). It is worth mentioning that pyrazines have been found in insects, including ants, and can function as both attractive and aversive pheromones (Guilford et al., 1987; Longhurst et al., 1978; Showalter et al., 2010; Xu et al., 2018). Our results highlight the importance of considering ant sampling location, even within a small study area. Previous work also suggests that Argentine ant colonies vary in iridomyrmecin abundance and this variation does not co-vary with invasion success (Salado et al., 2023) . While iridomyrmecin was detected in our extracts, it was not one of the top 25 metabolites found contributing to location differences in this study, and the identity of most compounds that promote the differences among our five sampling locations are unknown. In the same way, the identity of the compound(s) that previously invoked an avoidant response in *C. elegans* during chemotaxis assays (Alfonso et al., 2023) is still unknown too. Additionally, the lack of studies comparing variation among chemical profiles of Argentine ants and specific GC-MS databases for ant compounds in general made it difficult to complete the annotation of most compounds extracted in this study.

**Table 1.** The top 25 metabolites (and their normalized relative intensities averaged across each sampling location) contributing to the differences between sampling locations of the Argentine ants after GC/MS analysis. Five of 25 were tentatively annotated, and corresponding cosine values (reliability of annotation) and balance scores (quality of the deconvoluted mass spectra) are reported.

**Table:** 

Name	Cosine	Balance Score	Location 1	Location 2	Location 3	Location 4	Location 5
unknown 1			-0.51	1.49	-0.95	1.34	-1.38
unknown 2			-1.39	0.63	-1.03	2.07	-0.28
2,5-Dimethyl-3-(2-methylbutyl)pyrazine	0.87	72	-0.23	1.29	0.57	-0.48	-1.15
2-Isopentyl-3,5-dimethylpyrazine	0.94	100	-0.10	1.31	0.60	-0.51	-1.31
unknown 3			-1.68	0.67	-0.60	2.69	-1.08
unknown 4			-0.33	1.43	0.26	-0.47	-0.89
unknown 5			-0.92	0.78	-0.48	2.03	-1.41
unknown 6			-1.93	1.33	-1.43	1.39	0.64
unknown 7			-2.03	0.69	-0.64	2.14	-0.17
unknown 8			0.98	-1.31	1.18	0.54	-1.40
Heptadecanol-1	0.94	100	0.42	-0.53	1.19	0.31	-1.39
unknown 9			-0.73	-0.28	-0.54	2.72	-1.18
unknown 10			-0.16	1.00	0.57	0.01	-1.41
unknown 11			-0.17	1.13	0.64	-0.29	-1.31
unknown 12			0.19	0.57	1.16	-0.02	-1.90
Myristic acid	0.95	100	-0.41	0.42	1.30	0.35	-1.66
unknown 13			-0.18	1.03	0.62	-0.08	-1.39
unknown 14			-1.60	1.52	-0.37	0.73	-0.28
Palmitoleic acid	0.93	84	0.15	0.87	0.71	0.33	-2.07
unknown 15			-0.74	1.12	0.18	0.62	-1.18
unknown 16			-1.58	1.84	-1.51	1.03	0.22
unknown 17			-1.63	1.26	-1.03	0.97	0.43
unknown 18			0.12	0.78	0.90	-0.27	-1.53
unknown 19			-0.67	1.37	-0.11	0.92	-1.51
unknown 20			-0.74	0.93	0.35	0.49	-1.04

In summary, we report intraspecies chemical diversity of Argentine ants on the Stanford University campus. Our main motivation for this study was to explore possible explanations for the previous variability in the response of *C. elegans* to Argentine ant extract. Our findings suggest that environmental variability could have influenced the variation among chemical extracts used for testing in student *C. elegans* chemotaxis assays. However, it is unclear if the varied chemotaxis results occurred due to sampling location, or if Argentine ant chemical diversity changes from year to year, or both, and this may be tested in future work. For future studies, a guided fractionation employing High Performance Liquid Chromatography-Mass Spectrometry (HPLC-MS) could identify potential biomarkers associated with the chemotaxis response in *C. elegans* , or bioassays testing the specific effect of some compounds like iridomyrmecin or dolichodial, could be also beneficial. The more challenging task of identifying new compounds on Argentine ants (although not the goal of this study) would require large quantities of ants to isolate compounds and fully elucidate their structure using NMR and x-ray crystallography. More broadly, this body of work highlights how the repeated study of local diversity by student researchers in an undergraduate laboratory classroom setting, as shown here with natural ant chemical extracts and *C. elegans* chemotaxis assays, can lead to valuable basic science discoveries.

## Methods

*Ant extracts*

Argentine ant workers ( *Linepithema humile* ) were collected at five locations on the Stanford campus using insect aspirators (BioQuip). All colonies were more than 100 meters from each other, except for colony 1 and 3 (50 meters). Ants were kept cold until immobile at -20°C, and sorted on ice into glass vials of methanol (1:1, methanol:ant volume) (see (Bradon et al., 2023) ). Ants were pooled via colony location for each sample, and kept submerged in 1 mL of methanol for 24 hours at -80°C, and then the methanol was removed, placed in a new glass vial, and stored at -80°C until chromatographic analyses.

*Gas chromatography/mass spectrometry*

All ant samples were analyzed via gas chromatography coupled to mass spectrometry (GC/MS). Three samples were analyzed for each pooled location sample. Five blanks containing methanol were also analyzed. The system contained a GC-2030 Nexis, a single quadrupole mass spectrometer (QP-2020) and an autosampler SPL AOC-20i (Shimadzu, Nakagyo-ku, Kyoto, JP). A 1 μL sample was taken from each ant methanol sample and injected in splitless mode at 250°C. Separation of the sample occurred using a HP-5MS capillary GC column (30 m x 0.25mm x 0.25 μm, Agilent, Palo Alto, CA USA) at a flow rate of 1.0 mL/min. The temperature program began at 40°C for 3 minutes, increased to 100°C at a rate of 6°C/min, then to 200°C at 4°C/min and finally to 300°C at 20°C/min and held for 3 minutes. The ion source was set to 150°C and the interface temperature was set to 230°C. The mass spectrometry operated in full scan mode in a mass range of 40-500 m/z, scanning every 0.2 seconds.

*Data analysis*

The GC/MS files were converted to .CDF format and the Global Natural Products Social Molecular Networking (GNPS) open-access software was used to perform the deconvolution processing, which estimates feature intensities (Wang et al., 2016) . Molecular features with balance score < 60 were filtered out of analyses. The quality of the deconvoluted mass spectra is estimated through balance scores that range from 0–100 (higher balance scores indicate higher quality of the deconvoluted mass spectra). In the same way, to remove compounds eluted from the solvent or the chromatographic system, all those features with intensities >1000 in the majority of the blank samples were also removed. Metabolite data (peak intensities) were normalized by sum, log transformed, and scaled via Pareto scaling in Metaboanalyst (Pang, Lu, et al., 2024) . A principal component analysis was performed using Metaboanalyst (Pang, Xu, et al., 2024) to ask whether locations varied in chemical profiles across campus, and a PERMANOVA was used to test for differences between collection locations. A heatmap was also generated via Metaboanalyst, estimating Euclidean distances between the top 25 metabolites contributing to location differences, and performing a Hierarchical cluster analysis (HCA) using Ward’s method.

Subsequently, tentative metabolite annotation of these top 25 metabolites was performed in GNPS comparing our experimental data with mass spectra contained in reference libraries of natural products (NIST, Wiley, University of CORSICA, GNPS) (Table 1). From the GNPS library search process, the automatic annotation was manually reviewed comparing experimental mass spectra with those found in the reference libraries. Annotation reliability was supported by the respective cosine similarities that range from 0–1 (higher cosine indicates increased reliability of putative annotation) and balance scores.
